# Development of a semi-automated stewardship approach for prescriber-specific antibiotic consumption report cards

**DOI:** 10.1017/ash.2025.10068

**Published:** 2025-07-23

**Authors:** Shemual Tsai, Natalie Finch, Wesley Hoffmann, Shivani Patel, Evan Steere, Fadi Shehadeh, Muhammad Yasser Alsafadi

**Affiliations:** 1 Department of Pharmacy, Houston Methodist Hospital, Houston, TX, USA; 2 Department of Pharmacy, The University of Kansas Health System, Kansas City, KS, USA; 3 Department of Medicine, Houston Methodist Research Institute, Houston, TX, USA; 4 Department of Electrical Engineering and Computer Engineering, National Technical University of Athens, Athens, Greece; 5 Division of Infectious Diseases, Houston Methodist Hospital, Houston, TX, USA

## Introduction

Reporting antimicrobial consumption data to antimicrobial prescribers allows for benchmarking, transparency, and accountability. Individual prescriber report cards and peer comparisons have proven effective in reducing unnecessary antibiotic use in outpatient settings.^1,2^ However, the generation of individualized reports can be labor and time-intensive,which threatens sustainability. We describe our semi-automated approach to analyzing prescriber-specific antibiotic utilization and developing individualized report cards. Our initiative provides new perspectives on leveraging automation for antimicrobial stewardship (ASP) report cards and a novel peer comparison visualization. The source code is also shared for scalability and adaptability.

## Methods

This program was developed and implemented at a tertiary hospital with over 900 licensed beds, including facilities for cancer treatment and transplant centers. Infectious Diseases (ID) specialist prescribers who prescribed at least one antibiotic at our institution between January 1, 2024, and December 31, 2024, were included. These ID prescribers commonly enter antibiotic initiations, modifications, and discontinuations for their consulted patients directly into the electronic health record. The workflow for the initiative, including data processing, analysis, and individualized report card generation, was primarily performed using R. Report cards include personalized feedback, such as the top antibiotics contributing to days of therapy and spectrum score. Full technical components and details can be found in the supplementary files. The code is located under Research Transparency and Reproducibility. This project was undertaken as a Quality Improvement Initiative at Houston Methodist, and as such was not formally supervised by the Institutional Review Board.

## Results

**Figure 1.**
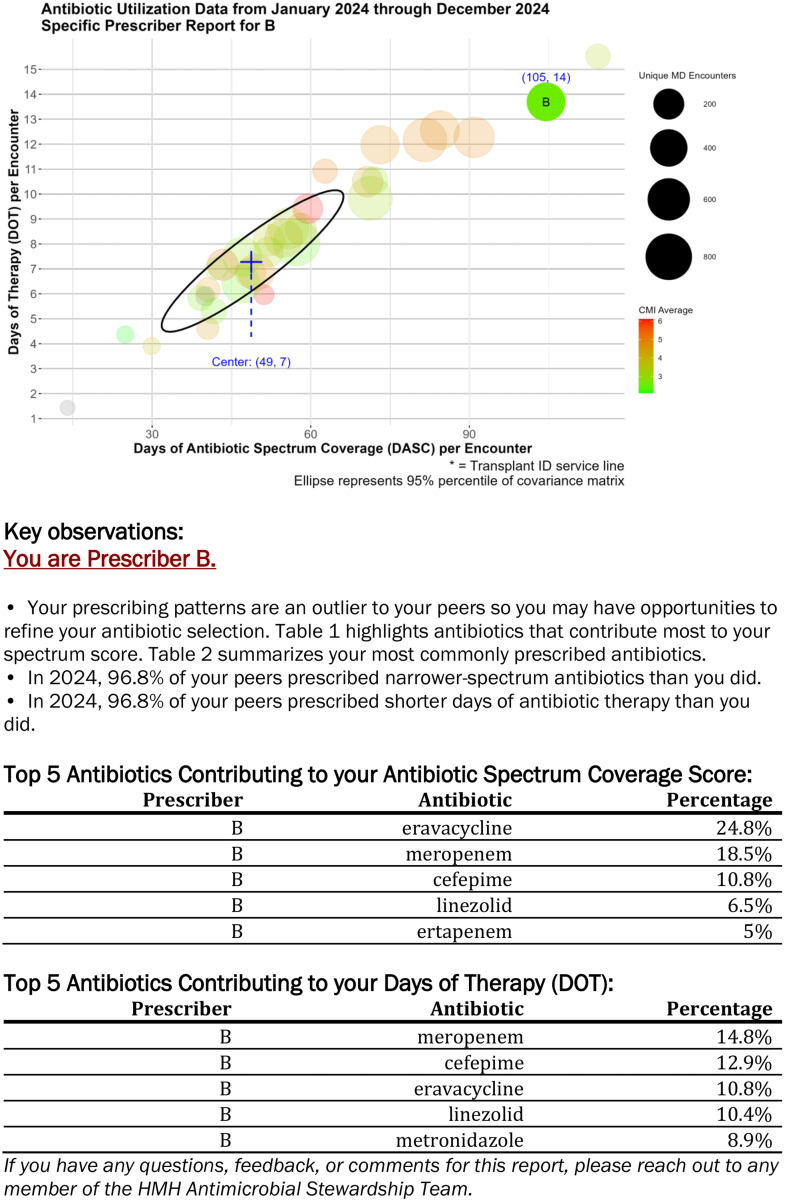
Example of page in the semi-automated generated report to ID prescribers. This page in the report includes a peer visualization element, as well as the top antibiotics contributing to the antibiotic spectrum coverage score and the number of days of therapy. In the visualization, each bubble represents a unique prescriber. The size of the bubble correlates to patient volume, the color correlates to patient acuity (case mix index), and the ellipse represents the 95% percentile of the covariance matrix. Prescribers with no case mix index data due to limited patient volume were colored gray. Prescribers were notified of their bubble in their individual report cards.

Thirty ID prescribers, including physicians, fellows, and advanced practice providers, were included in the analysis. Data were analyzed for 5,480 unique patients, 7,151 unique patient encounters, and 83,262 total days of therapy (DOT) among the ID prescribers. The median number of unique hospital patient encounters per prescriber was 204 (interquartile range 78–451) and the median case mix index was 3.05 (interquartile range 2.58–3.97). The reports, which were automatically generated and manually sent to prescribers, included a visualization of peer comparison and top antibiotics analysis, as shown in Figure 1.

## Discussion

This antimicrobial stewardship initiative introduces a novel approach for feedback and peer comparisons. To our knowledge, this is the first publication on semi-automated prescriber report cards that integrate multiple, objective data streams, including spectrum scores and patient acuity. Existing studies on inpatient antibiotic peer comparisons have not yet incorporated this level of automation, focused on ID prescribers, or addressed concerns that prescribing differences may be driven by patient acuity.^3–5^ Interestingly, two of our highest antibiotic utilizers, in both DASC/encounter and DOT/encounter, had a lower average case mix index than the median. Prior literature suggests a positive correlation between the case mix index and antibiotic utilization, and this may highlight opportunities for targeted feedback to these outlier prescribers.^6^

One key strength is the integration of automation into the workflow. Semi-automation reduces the time required for data processing and generating report cards. It also enhances consistency and reproducibility. Furthermore, it reduces manual errors, ensuring that any observed changes in prescribing habits result from true changes in prescriber patterns rather than errors in data collection or manipulation.

Another strength of this method is its objective assessment nature. Spectrum scores quantify antibiotic coverage, and the case mix index is routinely used at institutions nationwide and standardized in measurement.^7–9^ Objective feedback is provided with specific data on key antimicrobials that influence the prescriber’s score. This approach is also beneficial from an antibiotic stewardship perspective because it considers all prescribed antibiotics.

Our approach has several limitations. First, we analyzed prescription data as DOT/encounter versus DASC/encounter. This was a practical choice and requires further validation. Normalization through encounters can enable fair comparisons in peer feedback comparisons, given ID prescriber patient volumes at our institutions vary widely. Traditional metrics such as 1,000 patient days may be less meaningful at the individual prescriber level. Utilizing a surrogate of patient volume has also been utilized in other stewardship literature evaluating individualized prescriber peer comparisons.^10^ More importantly, our intention in sharing this initiative is not to advocate for a specific measure of antibiotic consumption but rather to present an adaptable and scalable approach.

Second, this method relies on administrative data, so we may miss encounters if antibiotics were continued in a setting that was not captured or if an ID prescriber saw a patient without prescribing any antimicrobials. Additionally, if patient care transfers occur without any changes in anti-infective orders, duration and spectrum may be attributed to the incorrect ID prescriber. It remains challenging to adequately attribute the spectrum and duration of therapy to the recommending consultant. Unfortunately, our database system is limited in the granularity of data to distinguish this. We maintained our methodology as we were aiming for an approximation of antibiotic utilization to facilitate a peer comparison visualization and do anticipate that actionable outlier trends would still emerge. Furthermore, the inclusion of 1 years’ worth of prescription data into the analysis helps partially overcome these limitations.

Ultimately, we aim for outlier scores to shift toward an inlier center, reflecting improved prescribing practices over time. We plan to continue to analyze the impact of these reports in conjunction with other antimicrobial stewardship initiatives on antibiotic utilization.

We present a semi-automated antimicrobial stewardship initiative designed to generate individualized prescriber feedback report cards, which is traditionally a resource-intensive task. The model’s scalable nature facilitates adaptability across various prescriber groups and facility types. This model will assist ASP teams to optimize stewardship workflows and support antimicrobial stewardship goals.

## Supporting information

10.1017/ash.2025.10068.sm001 Tsai et al. supplementary material Tsai et al. supplementary material

## Supplementary material

The supplementary material for this article can be found at https://doi.org/10.1017/ash.2025.10068

## Research Transparency and Reproducibility

The source code utilized for the workflow is openly accessible at https://github.com/shemtsai/semi-automated-report-cards
