# Supplementary material for: Development of a semi-automated stewardship approach for prescriber-specific antibiotic consumption report cards
Source: Antimicrob Steward Healthc Epidemiol. 2025 Jul 23;5(1):e162. doi: 10.1017/ash.2025.10068 (PMC12308614; doi:10.1017/ash.2025.10068)
Supplement: Tsai et al. supplementary material [file S2732494X25100685sup001.docx]

**Research Transparency and Reproducibility:** The source code utilized for the workflow is openly accessible at https://github.com/shemtsai/semi-automated-report-cards

**Methods**

*Analysis*

All anti-infective administration data from all ID prescribers across our institution were included. Non-systemic antibiotics were excluded. Each administered medication received an integer ASC score. To prevent false inflation, duplicate administrations of the same antibiotic by the same prescriber on the same date were assigned a score of zero. The DASC score was calculated as the sum of ASC scores per prescriber. To account for patient acuity, the case mix index was added as a surrogate marker for patient severity and complexity. The case mix index is a metric that incorporates variables such as patient diagnoses, comorbidities, procedures, and other coded documentation. The case mix index was calculated as the average of the sum of Medicare Severity-Diagnosis Related Group diagnosis-related group relative weight for each patient encounter at discharge. Any missing case mix index values were excluded from determining the average case mix index.

DOT were calculated by summing the instances when a patient received distinct systemic antibiotics from a unique prescriber on a specific calendar date. For example, if a patient received three doses of vancomycin and one dose of ceftriaxone from the same prescriber, on the same day, it was counted as two DOT. If a patient received two doses of vancomycin and one dose of ceftriaxone from one provider and a third dose of vancomycin from another prescriber, it was counted as two DOT for the first prescriber and one DOT for the second prescriber.

Patient encounters were defined by identifying the first instance of each unique ordering prescriber with each unique patient encounter for hospital admissions. Non-hospital admissions were not included. Each individual prescriber–patient encounter pair was counted as an individual patient encounter that then aggregated to represent the volume of patients seen per prescriber. For instance, if a prescriber ordered for any anti-infective at any one point during a patient’s encounter, this would have attributed as one patient seen by the prescriber. Since both DASC and DOT are cumulative metrics, they were then normalized by dividing the cumulative DASC and DOT, respectively, by the number of unique encounters for each prescriber.

The minimum covariance determinant method, which is a robust estimator of the mean and covariance matrix which accounts for statistical anomalies, was used to identify a robust center and outliers. The minimum covariance determinant method was implemented using the cov.mcd() function from the Modern Applied Statistics with S package in R. Outliers were defined as values that were outside the 95% confidence region of minimum covariance determinant inliers. The 95% confidence ellipse was then plotted for inlier values to illustrate the distinction between inliers and outliers. Outlier prescribers had their data reviewed manually to create additional opportunities for improvement based on objective measures of specific antibiotic prescribing habits compared to those of their peers. We targeted outlier prescribers with personalized feedback that included consumption percentiles for broad-spectrum antibiotics (e.g., meropenem and eravacycline).

*Workflow and Data Processing*

The workflow was primarily automated using R, with some manual steps incorporated (Figure 1). Data were manually extracted from the institution’s pharmacy surveillance database system, Vigilanz, which included antibiotic administration details such as route of administration, indication, and ordering provider. To account for individual patient acuity, case mix index values were retrieved from the Epic Clarity database. Data cleaning, processing, analysis, visualization, and report card generation were performed using R version 4.4.2. R packages used included readxl, MASS, ggplot2, officer, flextable, tidyr, dplyr, magrittr, writexl. R was utilized to promote generalizability, especially to ASP programs with limited coding experience, as it is an open-source language with a setup that does not require setting up virtual environments or managing package dependencies. Code development was assisted by an AI-based tool, ChatGPT (OpenAI) for optimization and troubleshooting.

Individualized prescriber report cards were generated via R and distributed via email. These report cards (an example is provided in the supplementary material) included objective data such as top five antibiotics contributing to the individual provider’s spectrum score and top five antibiotics contributing to DOT. They also highlighted improvement opportunities for outliers, stewardship best practices, and the latest ASP updates and initiatives to help prescribers make more informed decisions for future antibiotic prescriptions.

All data was collected, visualized, and integrated via scatterplots. The x-axis represented the spectrum score (DASC per encounter), while the y-axis represented the days of therapy (DOT per encounter). Prescriber names were de-identified using alphabetical labels. Infectious diseases physicians, specializing in immunocompromised patients, were marked with an asterisk to facilitate subgroup analyses. Bubble size and color gradient were used to represent the number of patient encounters and case mix index, respectively. The 95th percentile of the minimum covariance determinant inliers was also added to visualize outlier prescribers.

*Spectrum Scoring Criteria*

Spectrum scoring criteria utilized in the report were adapted from the DASC criteria established by Kakiuchi et al, with modifications based on limitations identified in existing literature on spectrum scores.^18,19^ These modifications were pre-established by the Antimicrobial Stewardship team, which consists of ID pharmacists and an ID physician.

Adjustments included updating scores by removing points previously assigned for *Staphylococcus aureus* coverage with aminoglycosides and restricting the analysis to systemic antibacterials. We expanded the spectrum scoring criteria for antimicrobial resistance to add a greater degree of clinical relevance. Inspired by the Infectious Diseases Society of America 2024 Guidance on the Treatment of Antimicrobial-resistant Gram-Negative Infections, we incorporated carbapenemase genes (KPC, IMP, VIM, NDM, OXA-48) and resistant pathogens, including carbapenem-resistant *Acinetobacter baumannii*, difficult-to-treat *Pseudomonas aeruginosa*, and *Stenotrophomonas maltophilia.^20^* Each antibiotic with activity against a listed gene or pathogen was assigned an additional point per target. The CRE definition was expanded to include non-carbapenemase-producing, carbapenem-resistant Enterobacterales to minimize duplicative scoring with the addition of carbapenemase genes.
